# Systemic redox status in lung cancer patients is related to altered glucose metabolism

**DOI:** 10.1371/journal.pone.0204173

**Published:** 2018-09-20

**Authors:** Katarzyna Zabłocka-Słowińska, Sylwia Płaczkowska, Anna Prescha, Konrad Pawełczyk, Monika Kosacka, Irena Porębska, Halina Grajeta

**Affiliations:** 1 Department of Food Science and Dietetics, Wroclaw Medical University, Wroclaw, Poland; 2 Diagnostics Laboratory for Teaching and Research, Wroclaw Medical University, Wroclaw, Poland; 3 Department and Clinic of Thoracic Surgery, Wroclaw Medical University, Wroclaw, Poland; 4 Department and Clinic of Pulmonology and Lung Cancers, Wroclaw Medical University, Wroclaw, Poland; University of South Alabama Mitchell Cancer Institute, UNITED STATES

## Abstract

Altered systemic redox status is often observed in lung cancer. However, detailed information on factors other, than smoking, which influence this perturbation is rather scarce. Elevated oxidative stress has been linked with disturbances in glucose metabolism before, but such associations have not been investigated in lung cancer. The aim of this study was to evaluate the relationship between systemic parameters of glucose metabolism and redox status in lung cancer patients (LC). Biochemical variables related to circulating glucose, i.e. glucose, insulin, c-peptide, fructosamine (FA), and glucose metabolism, i.e. β-hydroxybutyrate (BHB), lactate (LACT), non-esterified fatty acids (NEFAs), as well as redox status i.e. total antioxidant status (TAS) and total oxidant status (TOS) were determined for LC (n = 122) and control subjects (CS) (n = 84). HOMA-IR and the oxidative stress index (OSI) were calculated. LC patients had an altered redox status and glucose metabolism compared to CS. Positive correlations in LC were observed between TOS, OSI and circulating glucose as well as FA, while TAS positively correlated with BHB and NEFAs. In contrast, in metastatic LC, NEFAs and BHB positively correlated with OSI. Smoking status additionally stratified the observed relationships. In conclusion, we found that parameters related to circulating glucose or non-enzymatic glycation were correlated with oxidative stress (TOS and OSI), while metabolites such as BHB and NEFAs were correlated with antioxidant capacity (TAS). Metastasis prevalence and smoking seem to influence these correlations. However, the detailed mechanism of this relationship requires further research, in particular as regards the surprising positive correlation between NEFAs and TAS.

## Introduction

In lung cancer, as well as in many other site-specific cancers, the redox status is altered and depletion of antioxidant capacity is observed along with tumor progression [1–5]. Several mechanisms may induce this perturbation, e.g. smoking, increasing cancer-related chronic inflammation, decrease in dietary antioxidant intake, and cancer-related malnutrition with depletion of antioxidant proteins, as well as metabolic alterations [4–6]. During the past decade, a vast body of investigation has remodeled our understanding of human redox status and alterations regarding this phenomenon, including during chronic disease. However, still little is known about the associations between metabolic perturbations and redox status, especially among cancer patients [7,8].

As it has been shown many times, there is a close relationship between the risk of developing a range of solid tumors and altered glucose status [9–12]. On the other hand, glucose metabolism alterations became the first discovered metabolic abnormality during cancerogenesis [13]. Studies confirming an increase in disturbances in glucose metabolism along with cancer development and progression have been appearing for decades, and with great intensity in recent times. Milestones in research concerning glucose abnormalities during cancerogenesis have involved discoveries about the Cori cycle and Warburg effect, with many unresolved hypotheses and no clear explanation of these unusual phenomena [14,15], especially during cancer progression. Moreover, some other important explanations of the connections between glucose metabolism alterations and cancerogenesis exist, e.g. insulin and insulin-like growth factor (IGF) pathways targeting in cancer development [16] and increased expression of glucose transporters, especially glucose transporter 1 (GLUT1), in a variety of malignancies [17]. Some of these are closely related to redox signaling [18]. During lung cancer development and progression, alterations of glucose metabolism as well as depletion of total antioxidant capacity, show a deepening parallel. However, there have been no studies concerning the interactions between these two phenomena [5,19,20]. These alterations are unfavorable prognostic factors, finding potential relationships between them may enhance the management of lung cancer and create an opportunity for more personalized therapy, including the impact of these two alterations. Therefore, the aim of this study was to evaluate the relationship between systemic parameters of glucose metabolism and redox status in lung cancer patients.

## Subjects and methods

### Lung cancer patients and control subjects

Two hundred and six subjects took part in this study. Lung cancer patients, (n = 122) were recruited from the Lower Silesian Centre of Lung Diseases. Due to the possible effect of oncological treatment on glucose metabolism and redox status, patients were enrolled on the first day of admission to hospital, before any oncological treatment. The clinical stage of disease and metastases were evaluated based on chest computed tomography, PET-CT and ultrasonography of the abdominal cavity. CT/MRI of the central nervous system and scintigraphy of bone were performed if necessary. Bronchofiberoscopy was carried out routinely. In the case of enlarged lymph nodes of the mediastinum endobronchial ultrasound transbronchial needle aspiration (EBUS-TBNA) was performed. Negative EBUS results were verified in mediastinoscopy.

The control subjects (n = 84) consisted of healthy people recruited from the Wroclaw Universities of the Third Age and public offices. Exclusion criteria for the control group were as follows: cancers, metabolic disturbances, other pro-inflammatory diseases, neurological disorders and mental health issues.

From lung cancer patients, two groups were formed intentionally: with fasting glucose concentrations (GLC) ≤ 99mg/dL (n = 59), and GLC > 99 mg/dL (n = 63). Most lung cancer patients had diagnosed non-small cell lung cancer (NSCLC), and were diagnosed with different clinical stages of disease—about a third of them, regardless of glucose concentrations, were at stage I of disease. Metastasis (stage IV) was observed in a fifth of patients with normal glucose concentration, and about a tenth of lung cancer patients with >99 mg glucose/dL. Subjects differed in smoking status: the majority of control subjects were non-smokers (never or > 1 year smoking cessation) while in lung cancer patients—the majority were current or former smokers (≤ 1 year smoking cessation). Patients were also characterized in terms of the most commonly, chronically used drugs which could partially influence circulating glucose and insulin concentrations as well as parameters of glucose metabolism, e.g. NEFAs. In the case of control subjects, no chronic use of medicines was declared. Detailed data of lung cancer patients and control subjects are summarized in Table 1.

**Table 1:** Detailed baseline characteristics and nutritional status of lung cancer patients (n = 122) and control subjects (n = 84) [median (min-max)].

Variables	LC GLC ≤ 99 mg/dL (n = 59)	LC GLC > 99 mg/dL (n = 63)	CS GLC ≤ 99 mg/dL (n = 84)
**Gender, M/W [%]**	61.0/39.0^**a**^	57.6/42.4^**a**^	51.2/48.8^**a**^
**Age [years]**	65.0 (41.0–82.0)^**b**^	65.0 (55.0–82.0)^**b**^	60.0 (37.0–77.0)^**a**^
**NSCLC/SCLC/no data [%]**	71.2/11.9/16.9	63.5/3.2/33.3	-
**Clinical stage of disease; I/II/III/IV/no data [%]**	28.8/5.1/10.2/22.0/33.9	36.5/4.7/14.3/11.1/33.3	-
Smoking status current/former/no/no data [%]*	32.2/25.4/40.7/1.7	14.3/39.7/39.7/6.3	27.4/8.3/61.9/2.4
**Drugs: anti**–**DM/CVD/inhaled/NSAIDs/CNS/oral steroids [%]**	3.39/25.4/18.6/30.5/10.2/11.9	15.9/30.2/22.2/57.1/15.9/6.35	-
**Weight [kg]**	70.0 (43.0–146.0)^**b**^	77.0 (51.0–106.0) ^**ab**^	78.0 (52.7–118.0) ^**a**^
**BFP [%]**	27.1 (6.7–47.3)^**b**^	29.3 (7.1–50.0) ^**ab**^	31.8 (16.2–47.7) ^**a**^
**WHR**	0.94 (0.73–1.04)^**ab**^	0.95 (0.76–1.14)^**b**^	0.90 (0.73–1.08)^**a**^
**ALB [g/dL]**	3.66 (2.83–4.35)^**b**^	3.80 (2.70–5.15)^**b**^	4.20 (1.62–5.16) ^**a**^
**Energy intake [kcal/d]**	2131.0 (1058.0–3323.9)^**a**^	2531.2 (699.1–4394.1)^**b**^	1913.8 (741.6–3980.2) ^**a**^
**Total carbohydrate intake [g/1000 kcal]**	141.9 (58.4–196.0)^**a**^	132.7 (98.3–179.6) ^**a**^	137.9 (91.6–199.9) ^**a**^
**Digestible carbohydrates [g/1000 kcal]**	130.9 (52.9–178.5)^**a**^	122.5 (89.0–164.2) ^**a**^	125.5 (82.6–182.4) ^**a**^
**Dietary fiber intake [g/1000 kcal]**	11.0 (5.5–19.8)^**ab**^	9.7 (6.3–17.9)^**b**^	11.5 (5.8–23.2) ^**a**^

LC—lung cancer patients; CS—control subjects; GLC—glucose concentration; M—men; W—women; anti—DM—antidiabetic agents; CVD—agents used for cardiovascular diseases; inhaled agents—glucocorticoids and/or beta2-agonists; NSAIDs—non-steroidal anti-inflammatory drugs; CNS—central nervous system drugs; BFP—body fat percentage; WHR—waist-hip ratio; ALB—albumin concentration; NSCLC—non-small cell lung cancer; SCLC—small cell lung cancer; differences between groups are presented with different superscripts (ANOVA test performed on transformed data, see statistical analysis);

* indicates significant differences in smoking status (Chi-square test)

### Methods

#### Anthropometric measurements and dietary intake

Anthropometric parameters as well as energy and nutrient intakes were used for the nutritional status assessment of participants. Baseline anthropometric parameters: weight, body fat percentage (BFP) and waist-hip ratio (WHR) were measured. The percentage of body fat was determined using a Body FAT Monitor (Omron BF 306, Japan). WHR was calculated as the ratio of waist and hip circumference. Waist circumference was measured at the level of the umbilicus, and hip circumference at the trochanter levels. All anthropometric measurements were performed twice.

Dietary data were gathered using three 24-h dietary recalls by a trained interviewer. In the case of lung cancer patients this took place on the first day of admission to hospital. To assess information about the portion size of food products, the Album of photographs of food products and dishes (National Food and Nutrition Institute, Warsaw, Poland) was used [21]. All dietary recalls were analyzed using Dieta 5.0 (National Food and Nutrition Institute, Warsaw, Poland).

#### Blood collection and preparation

The day after patient admission to hospital, after overnight fasting, blood samples were collected and serum was separated. All material was stored at -80°C until analysis. The study protocol was approved by the Ethics Commission of Wroclaw Medical University (approval no. 540/2013), and the study was conducted according to the principles expressed in the Declaration of Helsinki. All participants provided written consent for taking part in the research.

#### Parameters measured in serum, related to systemic redox status

*Total antioxidant status (TAS)*: Total antioxidant status (TAS) was measured using the commercial TAS kit (Randox Laboratories, UK). The analysis performed in serum is based on incubation of 2,2′-azino-di-(3-ethyl-benzthiazoline sulfonate) (ABTS) with a peroxidase (metmyoglobin) and H_2_O_2_ to produce the radical cation ABTS^+^. This cation has a relatively stable blue-green color which is measured at 600 nm. The blood antioxidants cause the cationic neutralization and the disappearance of colour, which is measured. The assay is calibrated with Trolox and results are expressed in terms of mmol/L Trolox. Analyses were performed on an auto-analyzer (Konelab 20i Thermo Fisher Scientific, USA).

*Total oxidant status (TOS)*: Total oxidant status (TOS) was measured as described by Erel [22]. Using this method, the oxidants present in the sample oxidize the ferrous ion-o-dianisidine complex to the ferric ion. The ferric ion produces a colored complex with xylenol orange in an acidic medium. The color intensity, which is measured spectrophotometrically (560 nm), is related to the total amount of oxidant molecules present in the sample. Briefly, the first absorbance is measured after mixing serum (35 μl) with reagent 1 (orange 150 μM, NaCl 140 mM and glycerol 1.35 M in 25 mM H_2_SO_4_ solution, pH 1.75) as a sample blank, the second one after adding reagent 2 (ferrous ion 5 mM and *o*-dianisidine 10 mM in 25 mM H_2_SO_4_ solution) when the reaction reaches a plateau (3 min after mixing). The assay was calibrated with hydrogen peroxide and the results were expressed in terms of μmol H_2_O_2_ equivalent/L of serum. TOS measurements were performed on a UV-6300PC spectrophotometer (VWR International, China).

*Oxidative stress index (OSI)*: The TOS: TAS ratio was used as the oxidative stress index (OSI), and calculated as follows:

$$\text{OSI}\;(\text{arbitrary}\;\text{units})=[(\text{TOS},\;\mu \text{mol}\;{\text{H}}_{2}{\text{O}}_{2}/\text{L})\;/\;(\text{TAS}\;\text{mmol}\;\text{Trolox}\;\text{equiv}./\text{L})]$$

[23].

#### Parameters measured in serum related to glucose metabolism

*Glucose*: Glucose concentration (GLC) was quantified with a commercial kit, cat. no. 981780 (Thermo Fisher Scientific, USA), based on an enzymatic coupled assay using the glucose oxidase and peroxidase method (GOD-POD). Glucose is oxidized to D-gluconate by a glucose oxidase with the formation of an equimolar amount of hydrogen peroxide. In the presence of peroxidase, 4-aminoantipyrine and phenol are oxidatively coupled by hydrogen peroxide to form a quinone imine dye, colored red. The intensity of color in the reaction is measured at 510 nm and is proportional to the glucose concentration in the sample. Assays were performed on an auto-analyzer (Konelab 20i Thermo Fisher Scientific, USA).

*Insulin*: Insulin concentration (INS) was determined with DRG Insulin ELISA (DRG Instruments GmbH, Germany), cat no. EIA-2935. The test is based on the sandwich principle. Serum of subjects was incubated in the coated well with enzyme conjugate, which is an anti-insulin antibody conjugated with biotin. Next, the streptavidin peroxidase enzyme complex binds to the anti-insulin antibody. The insulin standards are calibrated against international World Health Organization (WHO) approved reference material NIBSC 66/304. The complex is proportional to the concentration of serum insulin concentration which is expressed by the intensity of the color complex. The color intensity of the reaction was measured at 450 nm using the microplate reader Multiscan GO (Thermo Scientific, USA).

#### Homeostasis Model Assessment for Insulin Resistance (HOMA-IR)

HOMA-IR was calculated using the equation:

HOMA-IR=fastinginsulin(μU/mL)xfastingglucose(mg/dL)/405

[24].

#### C-peptide

C-peptide concentration (C-PEP) was determined with DRG C-Peptide ELISA (DRG Instruments GmbH, Germany), cat no. EIA-1293. The test is based on the principle of competitive binding. Endogenous C-peptide of the subject’s sample competes with a C-peptide horseradish peroxidase conjugate for binding to the coated antibody. After incubation the unbound conjugate is washed off. The amount of bound peroxidase conjugate is inversely proportional to the concentration of C-peptide. The C-peptide standards are calibrated against international WHO approved reference material IRR c-peptide, code 84/510. The color intensity of the reaction was measured at 450 nm using the microplate reader Multiskan GO (Thermo Fisher Scientific, USA).

#### Fructosamine

Fructosamine concentration (FA) was measured using a colorimetric assay kit (LT-SYS; Labor +Technik, Berlin, Germany), cat. no. LT-FR 0026. This method was based on the ability of ketoamines to reduce nitrotetrazolium-blue to formazan in an alkaline solution. The rate of formation of formazan is directly proportional to the concentration of fructosamine. Due to the disruption of protein metabolism in cancer patients, and significantly lower albumin concentration in lung cancer patients compared to healthy subjects, we decided to express fructosamine as fructosamine/g of albumin (FA/g ALB). Measurements were performed photometrically at 546 nm on an auto-analyzer (Konelab 20i Thermo Fisher Scientific, USA).

#### Albumin

Albumin concentration (ALB) was measured using a commercial assay kit, cat. no. 981767 (Thermo Electron Corporation, Finland) based on the colorimetric method with bromocresol green. When albumin reacts with bromocresol green, colored products are formed. The test is based on the formed color intensity at 600 nm. Analyses were performed on an auto-analyzer (Konelab 20i Thermo Fisher Scientific, USA).

This parameter was measured for converting fructosamine concentration from μmol/L to μmol/1g of ALB.

#### Lactates

Lactate concentration (LACT) was determined using Lactate FS, cat. no. 1 4001 99 10 021 (DiaSys Diagnostic Systems GmbH). The principle of this method is based on spectrophotometrically measured lactate in a sample, by using lactate dehydrogenase to oxidize lactate in the presence of nicotinamide adenine dinucleotide (NAD) to pyruvate. Light at 340 nm is used to measure the dihydronicotinamide adenine dinucleotide (NADH) formed. This is related to the lactate concentration. Measurements were performed on an auto-analyzer (Konelab 20i Thermo Fisher Scientific, USA).

#### Non-esterified fatty acids

Concentrations of non-esterified fatty acids (NEFAs) were determined using the commercial kit NEFA FS cat. no. 1 5781 99 10 935 (DiaSys Diagnostic Systems GmbH, Germany). Using this method NEFAs are converted into Acyl-CoA in the presence of Acyl-Co synthetase. Acyl-Co is then oxidized to H_2_O_2_ with Acyl-Co oxidase. A red product is formed from H_2_O_2_ in the presence of peroxidase. The intensity of the red color is measured at 546 nm, and is directly proportional to the amount of NEFAs present in the serum. Due to depletion of serum reserves, we measured NEFAs only in 37 patients out of LC with GLC >99 mg/dL and 26 out of LC ≤ 99mg/dL. Concentrations of NEFAs were measured on an auto-analyser (Konelab 20i ThermoScientific, USA).

#### β-hydroxybutyrate

Concentrations of β-hydroxybutyrate (BHB) were measured with the commercial kit β hydroxybutyrate FS cat. no. 1 3701 99 10 930 (DiaSys Diagnostic Systems GmbH, Germany). This method is based on the oxidation of BHB to acetoacetate with a concomitant reduction of NAD^+^ to NADH. Diaphorases catalyze the oxidation of NADH by nitroblue tetrazolium (NBT). The intensity of the blue color of reduced NBT is directly proportional to the amount of BHB. Analyses were performed on an auto-analyzer (Konelab 20i Thermo Fisher Scientific, USA).

#### Statistical analysis

The data were analyzed using Statistica 12, PL (StatSoft). To evaluate the differences in distribution of carbohydrate metabolism and redox status parameters between lung cancer and the control group, and according to carbohydrate metabolism perturbations, the ANOVA test was performed after logarithmic transformation due to the, in part, non-parametric data. Tukey’s post hoc test was performed for intergroup comparison of data. Missing data were replaced by the respective group average. The correlations between parameters related to carbohydrate metabolism and redox status parameters were assessed using Pearson correlation coefficient. The chi-square test was used for intergroup comparisons of clinical type, stage of disease and smoking status. For all statistical procedures, the significance level was considered to be < 0.05.

## Results

Results concerning baseline characteristics and nutritional status of lung cancer and control participants are summarized in Table 1. Lung cancer patients with GLC ≤ 99 mg/dL had significantly lower weight and BFP while lung cancer subjects with GLC > 99 mg/dL had a significantly higher WHR compared to control subjects. All lung cancer patients, regardless of glucose concentration, had significantly lower serum ALB in comparison with CS. Lung cancer patients with GLC > 99 mg/dL provided significantly higher amounts of energy in their diets compared to patients with GLC ≤ 99 mg/dL as well as to control subjects, and provided significantly less fiber in their diet compared with control subjects. Patients, regardless of glucose concentration, were sex- and age-matched, and were not differentiated by clinical stage of disease or type of lung cancer.

Systemic parameters related to glucose metabolism and redox status in control subjects (CS) and lung cancer patients (LC), distributed according to glucose concentration, are presented in Table 2. Lung cancer patients with GLC > 99 mg/dL had significantly higher serum INS, C-PEP and HOMA-IR levels compared to patients with GLC ≤ 99 mg/dL, or with control subjects. No differences in serum GLC, INS, C-PEP or HOMA-IR levels were observed between patients with GLC ≤ 99 mg/dL and control subjects. Statistical analysis revealed significantly higher serum BHB and LACT in lung cancer patients, regardless of glucose concentrations, compared to control subjects. NEFAs differed only between sub-groups of lung cancer patients with GLC > and ≤ 99 mg/dL, not when compared to control subjects. We did not find any significant differences in FA/g ALB between any analyzed groups.

**Table 2:** Systemic parameters related to glucose metabolism and redox status in lung cancer patients with glucose ≤ 99 mg/dL (n = 59), glucose > 99 ng/dL (n = 63) and control subjects (n = 84) [median (min-max)].

Parameters	LC GLC ≤ 99 mg/dL	LC GLC > 99 mg/dL	CS GLC ≤ 99 mg/dL
**parameters related to glucose metabolism**			
**GLC [mg/dL]**	87.5 (57.0–97.0)^**a**^	132.0 (112.0–296.0)^**b**^	86.0 (69.2–99.0)^**a**^
**INS [μIU/mL]**	9.76 (1.16–112.7)^**a**^	22.90 (3.75–115.5)^**b**^	10.24 (3.81–28.2) ^**a**^
**HOMA-IR [arbitrary unit]**	2.16 (0.24–27.0) ^**a**^	7.66 (1.33–81.6)^**b**^	2.10 (0.86–28.21) ^**a**^
**C-PEP [ng/mL]**	2.82 (0.13–12.2) ^**a**^	5.08 (0.25–12.4)^**b**^	2.52 (0.62–9.64) ^**a**^
**BHB [mmol/L]**	0.08 (0.03–1.11)^**b**^	0.1 (0.02–2.57) ^**b**^	0.04 (0.02–0.39) ^**a**^
**FA [μmol/g ALB]**	8.58 (6.23–12.2) ^**a**^	8.50 (6.15–17.1) ^**a**^	8.52 (0.01–20.9) ^**a**^
**LACT [mmol/L]**	2.85 (0.74–4.67) ^**b**^	2.5 (0.95–3.22) ^**b**^	1.87 (1.09–3.88) ^**a**^
**NEFAs [mmol/L]**	0.48 (0.25–3.30) ^**a**^	0.61 (0.27–1.46) ^**b**^	0.55 (0.31–1.05) ^**ab**^
**parameters related to redox status**			
**TAS [mmol Trolox equiv./L]**	1.58 (0.96–2.34)^**b**^	1.60 (1.12–2.55)^**b**^	1.71 (1.20–2.10) ^**a**^
**TOS [μmol H**_**2**_**O**_**2**_ **equiv./L]**	3.07 (1.00–49.6) ^**a**^	3.86 (0.89–74.8) ^**b**^	2.94 (0.51–49.3) ^**a**^
**OSI [arbitrary unit]**	1.96 (0.74–28.2) ^**a**^	2.64 (0.67–51.9) ^**b**^	1.77 (0.34–32.96) ^**a**^

LC—lung cancer patients; CS—control subjects; GLC—glucose concentration; INS—insulin concentration; HOMA-IR—homeostasis model assessment—insulin resistance; C-PEP—C-peptide concentration, BHB—β-hydroxybutyrate concentration; LACT—lactate concentration; FA—fructosamine concentration; NEFAs—non-esterified fatty acids concentration; TAS—total antioxidant status; TOS—total oxidant status; OSI—oxidant status index; differences between groups are presented with different superscripts (ANOVA test performed on transformed data, see statistical analysis)

Assessment of redox status showed significant differences in serum TAS, TOS and OSI between control subjects and lung cancer patients. Serum TAS of control subjects was significantly higher compared to lung cancer patients with GLC > 99 mg/dL as well as with GLC ≤ 99 mg/dL. However, statistical analysis of TOS and OSI results showed significant differences between control subjects and patients with lung cancer, but only those with GLC > 99 mg/dL.

Results concerning systemic parameters of glucose metabolism, and redox status after distribution of lung cancer patients according to metastasis prevalence are presented in Table 3. We did not find any significant differences in redox status parameters or parameters related to glucose metabolism, except for a significantly lower serum GLC and, HOMA-IR level, and higher LACT in metastatic patients compared to non-metastatic ones. We also statistically analyzed parameters of glucose metabolism and redox status in lung cancer patients, across four clinical stages of disease. There were no significant differences (checked with ANOVA test) in parameters between patients in particular stages of disease (S1 Table).

**Table 3:** Systemic parameters related to glucose metabolism and redox status in metastatic (n = 20) and non-metastatic (n = 61) lung cancer patients; median (min-max).

Parameters	Metastatic LC (35% with GLC > 99 mg/dL)		Non-metastatic LC (42.6% with GLC > 99 mg/dL)
**parameters related to glucose metabolism**			
**GLC [mg/dL]**		91.0 (64.0–230.0)^a^	111.0 (57.0–255.0)^a^
**INS [μIU/mL]**		9.29 (3.13–89.5)	19.9 (1.16–112.7)
**HOMA-IR [arbitrary unit]**		2.58 (0.49–41.3)^a^	5.25 (0.24–68.3)^a^
**C-PEP [ng/mL]**		2.91 (0.90–9.36)	3.71 (0.13–12.2)
**BHB [mmol/L]**		0.10 (0.03–1.05)	0.10 (0.02–1.20)
**FA [μmol/g ALB]**		8.54 (6.23–17.1)	8.44 (6.15–14.5)
**LACT [mmol/L]**		3.13 (1.50–4.48)^a^	2.40 (0.95–4.67)^a^
**NEFAs [mmol/L]**		0.56 (0.28–0.98)	0.51 (0.25–1.46)
**parameters related to redox status**			
**TAS [mmol Trolox equiv./L]**		1.57 (1.12–2.01)	1.62 (0.96–2.55)
**TOS [μmol H**_**2**_**O**_**2**_ **equiv./L]**		3.20 (1.00–25.5)	3.61 (0.89–74.8)
**OSI [arbitrary unit]**		1.89 (0.74–17.61)	2.48 (0.66–51.9)

LC—lung cancer patients; GLC—glucose concentration; INS—insulin concentration; HOMA-IR—homeostasis model assessment—insulin resistance; C-PEP—C-peptide concentration; BHB—β-hydroxybutyrate concentration; FA—fructosamine concentration; LACT—lactate concentration; NEFAs—non-esterified fatty acids concentration; TAS—total antioxidant status; TOS—total oxidant status; OSI—oxidant status index

^**a**^–statistically significant differences (Mann Whitney U-test; p<0.05)

Correlation analysis revealed significant relationships between systemic parameters regarding glucose metabolism and redox status, different—in lung cancer patients and control subjects (Table 4). Circulating glucose concentration positively correlated with TOS (R = 0.25, *p = 0*.*005*)—in all lung cancer patients and these associations were stronger in subgroups of former smokers as well as in metastatic patients. Similar associations were observed between OSI and circulating glucose: there was a positive correlation (R = 0.29; p = *0*.*001*) between these two parameters in all lung cancer patients, which was much stronger (R = 0.60; p = *0*.*005*) in the subgroup with metastasis. However, distribution of patients according to glucose concentration revealed that the association occurred only in patients with GLC > 99 mg /dL (R = 0.27; p = *0*.*033*). Analogous relationships were observed for fructosamine—this parameter significantly, positively correlated with TOS (R = 0.20; p = *0*.*031*) and OSI (R = 0.24; p = *0*.*007*) in all lung cancer patients and relationships were much more pronounced in metastatic patients (R = 0.54; p = *0*.*015* and R = 0.63; p = *0*.*003*, respectively). Insulin negatively correlated (R = -0.26; p = *0*.*048*) with TAS but only in non-metastatic patients, while HOMA-IR correlated positively with TOS in former smoking patients (R = 0.33; p = *0*.*037*). No correlation was observed between c-peptide and parameters of redox status in the lung cancer group. B-hydroxybutyrate positively correlated with TAS, but only in the following subgroups of lung cancer patients: with GLC ≤99 mg/dL (R = 0.33; *p = 0*.*012*), non-smoking ones (R = 0.45; p = *0*.*001*) and non-metastatic patients (R = 0.49;p<*0*.*001*). Interestingly, after separating patients with metastasis, we found a strong positive correlation between BHB and OSI (R = 0.48; p = *0*.*033*). Surprisingly, NEFAs positively correlated with TAS (R = 0.21; *p* = *0*.*024*) in all lung cancer patients, and the demonstrated association was further intensified in non-smoking and non-metastatic patients (R = 0.49; p<*0*.*001* and R = 0.50; p<*0*.*001*, respectively). In metastatic ones we found a strong positive correlation between NEFAs and OSI (R = 0.49; p = *0*.*029*).

**Table 4:** Significant correlations observed between systemic parameters related to glucose metabolism and redox status in lung cancer patients and control subjects.

Parameters related to glucose metabolism	TAS	TOS	OSI
	R; *p* (group)	R; *p* (group)	R; *p* (group)
**GLC**	NS	**0.25; *0*.*005*** (all LC)	**0.29; *0*.*001*** (all LC)
		**0.31; *0*.*016*** (non-meta LC)	**0.27; *0*.*033*** (LC > 99mg GLC/dL)
		**0.45; *0*.*004*** (former smoking LC)	**0.33; *0*.*009*** (non-meta LC)
		**0.51; *0*.*023*** (meta LC)	**0.60; *0*.*005*** (meta LC)
**FA/g ALB**	NS	**0.20; *0*.*031*** (all LC)	**0.24; *0*.*007*** (all LC)
		**0.28; *0*.*026*** (LC > 99mg GLC/dL)	**0.26; *0*.*046*** (non-meta LC)
		**0.36; *0*.*024*** (former smoking LC)	**0.33; *0*.*009*** (LC > 99mg GLC/dL)
		**0.54; *0*.*015*** (meta LC)	**0.63; *0*.*003*** (meta LC)
**INS**	**-0.26; *0*.*048*** (non-meta LC)	NS	NS
**HOMA—IR**	NS	**0.33; *0*.*037*** (former smoking LC)	NS
**BHB**	**0.33; *0*.*012*** (LC ≤ 99 mg GLC/dL)	NS	**0.48; *0*.*033*** (meta LC)
	**0.45; *0*.*001*** (LC non-smoking)		
	**0.49; <*0*.*001*** (non-meta LC)		
**NEFAs**	**0.21; *0*.*024*** (all LC)	NS	**0.49; *0*.*029*** (meta LC)
	**0.33; *0*.*007*** (LC > 99mg GLC/dL)		
	**0.49; <*0*.*001*** (non-smoking LC)		
	**0.50; <*0*.*001*** (non-meta LC)		
**C-PEP**	**0.28; *0*.*010*** (all CS)	NS	NS
	**0.52; *0*.*010*** (smoking CS)		
**LACT**	**0.36; *0*.*001*** (all CS)	**0.30; *0*.*028*** (non-smoking CS)	NS
	**0.49; <*0*.*001*** (non-smoking CS)		

TAS—total antioxidant status; TOS—total oxidant status; OSI—oxidative stress index; GLC—glucose concentration; FA—fructosamine concentration; INS—insulin concentration; HOMA-IR—homeostasis model assessment—insulin resistance; C-PEP—c-peptide concentration; BHB—β-hydroxybutyrate concentration; LACT—lactate concentration; NEFAs—non-esterified fatty acids concentration; ALB—albumin concentration; NS—non-significant; All LC—all lung cancer patients; CS—control subjects; LC >99mg GLC—lung cancer patients with elevated glucose concentration; LC ≤99 mg GLC—lung cancer patients with normal glucose concentration; meta LC—lung cancer patients with metastasis; non-meta LC—lung cancer patients without metastasis; smoking LC—current smoking lung cancer patients; former smoking LC—lung cancer with ≤ 1 year smoking cessation; non-smoking LC—never smoking lung cancer patients or > 1 year smoking cessation All CS—all control subjects; former smoking CS—control subjects with ≤ 1 year smoking cessation; non-smoking CS—never smoking control subjects or > 1 year smoking cessation

In control subjects C-peptide positively correlated with TAS (R = 0.28; p = *0*.*010*) and this relationship was intensified by smoking (R = 0.52; p = 0.010). Lactate revealed an association with TAS also only in control subjects and in subgroup of non-smoking ones (R = 0.36; p = *0*.*001* and R = 0.49; p<*0*.*001*, respectively). Interestingly, this parameter also positively correlated (R = 0.30; p = *0*.*028*) with TOS in the subgroup of non-smoking controls.

## Discussion

Large studies confirmed that systemic oxidative stress is associated with pathogenesis of many site-specific cancers, including lung cancer [3,25–27]. However there has been no advanced research indicating that metabolic alterations may influence the depletion of systemic antioxidant activity during cancerogenesis, and therefore contribute at least partially to oxidative stress, regardless of the tumor process itself. In light of previous papers reporting that systemic redox balance is disrupted during oncological treatment [28,29,30] the knowledge of the influence of metabolic disorders on redox status appears to be important also for the effectiveness of oncological treatment. Among metabolic disorders, alterations in glucose metabolism seem to be key in terms of tumorigenic properties as well as control of redox status in the tumor milieu, and much research has been devoted to investigating particular metabolic pathways that explain this phenomenon [31,32]. However, little is known about systemic changes in the context of glucose metabolism and oxidative stress in cancer patients. In lung cancer, the prevalence of alterations in glucose metabolism is high in general and is a consequence of cancerogenesis rather than an independent metabolic disorder that increases the risk of lung cancer [33,34,35]. Indeed, recent experimental research has revealed that lung cancer patients are characterized by orchestrated activation of glucose absorption and metabolism towards the anaerobic pathways [36]. Based on above-mentioned data, we attempted to find a link between systemic glucose metabolic alterations and redox status in lung cancer patients.

In this study increased serum insulin, C-peptide concentrations and HOMA-IR value observed in lung cancer patients were evidently direct consequences of elevated circulating glucose concentration. The lack of differences in the above parameters between lung cancer patients with glucose concentrations in the reference range and control subjects indicates that lung cancerogenesis did not influence insulin and c-peptide concentration by itself without alterations in glucose concentration. In the study by Petridou et al. [37], lung cancer patients had significantly higher HOMA-IR levels compared to healthy controls; however, the authors did not select patients with a normal glucose concentration, and their results only confirm that lung cancer patients had a high prevalence of increased insulin resistance, not that lung cancerogenesis directly influences it. Similar results, but concerning c-peptide, were observed by Zhang et al. [38]. Additionally, the authors distributed lung cancer patients into two groups according to prevalence of type 2 diabetes mellitus (T2DM), and found that patients with T2DM had significantly higher c-peptide concentrations compared to those without T2DM. Our results concerning serum c-peptide concentrations in studied sub-groups revealed that lung cancer patients with normal glucose concentrations as well as control subjects had c-peptide concentrations comparable to those observed in the Zhang et al. [38] study for patients without T2DM. The data presented above unequivocally confirm the lack of an independent and direct impact of lung cancerogenesis on insulin, c-peptide and HOMA-IR concentrations and indicate that disturbances in these parameters result from alterations in circulating glucose.

The high concentration of NEFAs observed in lung cancer patients in the study is also linked with concentration of glucose. Impaired metabolism of glucose towards glucose intolerance is mainly demonstrated via a decrease in passage of glucose through the plasma membrane of peripheral and hepatic cells. This type of cellular “starvation” is covered by release of fatty acids from triglycerides which in turns results in higher NEFA concentrations [39]. On the other hand, for decades it has gained widespread acceptance that high concentrations of NEFAs can contribute to insulin resistance, e.g.by the toxic influence on pancreatic β-cells and thus impaired insulin secretion and peripheral sensitivity to insulin, and also by increasing activation of proinflammatory cytokines as well as oxidative stress [40]. Results obtained in this study indicated that the increase in circulating NEFAs in lung cancer patients was associated with elevated glucose concentration. However, differences in NEFAs concentrations were observed only when compared between lung cancer patients with normal and elevated glucose concentrations, not between lung cancer patients with elevated glucose and control subjects, which suggests that besides circulating glucose, cancerogenesis in fact also might impair NEFA concentrations. The observed slight tendency (without statistical significance) to a lower NEFAs level in lung cancer patients with normal glucose concentration in comparison with control subjects was probably a consequence of cancer-related worse nutritional status and significantly lower body fat percentage. It has been shown that in cancer-related malnutrition the triacylglycerol/fatty acid substrate cycle is altered, and these disturbances might interfere with NEFA effects on glucose metabolism [41].

Elevated β-hydroxybutyrate which has been observed in lung cancer patients, seemed to result from carcinogenesis rather than from alterations in circulating glucose. The increase in β-hydroxybutyrate in lung cancer patients could result from cancer-related malnutrition and/or, which is most likely, from alteration in glucose metabolism, characteristic for tumorgenesis [42]. Ketone bodies are substrates for effective energy production in healthy cells under physiological conditions, but cancer tissues cannot efficiently utilize them. This is a direct effect of widespread mitochondrial pathology in most cancers, including lung cancer [43], manifested by decreased mitochondrial numbers, abnormal ultrastructural morphology, swelling and cristolysis of these organelles, mtDNA mutations and many other effects [44]. Inability to utilize ketone bodies may result in their elevated concentrations in cancer patients as observed in our study. However, further studies are needed to confirm this hypothesis, due to the knowledge gap in this area. Another possible mechanism of elevated ketone bodies, known as “two-compartment tumor metabolism” is related to the highly upregulated enzymes required for ketone body production within cancer associated fibroblasts. This hypothesis was confirmed by Martinez-Outschoorn et al. [45] who showed that enzymes associated with ketone body production were preferentially expressed in breast tumor stroma.

In this study we found elevated lactate concentrations in lung cancer patients, irrespective of circulating glucose, but we did not observe lactate levels > 5 mmol/L, which is one of the diagnosis criteria of lactate acidosis [46]. This may suggest that lactate concentrations are affected by cancer development, in agreement with the study by Holroyde et al. [47], where elevated lactate as well as its overproduction was observed in colorectal cancer patients compared to healthy controls. Several hypotheses concerning the mechanism of elevated lactate production and concentrations accompanying oncological disease have been proposed, including: extensive accumulation of glucose in neoplastic cells and therefore overproduction of lactate (including from glutamine and serine), activation of hypoxia inducible factor (HIF), changes in the activity of enzymes related to glucose metabolic pathways, liver dysfunction due to cancer metastases and ischemia in cancer tissues [46]. Moreover, intensification of lactate synthesis increases the risk of metastases in different cancers including head-and-neck, breast, cervical cancer and many others [48–50]. Our results indicating significantly higher concentrations of lactate in metastatic than non-metastatic patients confirmed data presented in the above-cited studies. Metastasis of tumor is stimulated by secretion of hyaluronan by fibroblasts in stroma associated with tumor as well as by overexpression of CD44, a major cell hyaluronan receptor—both of which are induced by lactates. Alternative mechanisms of lactate-stimulation tumor cells motility are up-regulation of vascular endothelial growth factor (VEGF) activity, stimulation of IL-8-dependent angiogenesis, activation of β1-integrins, promotion of the TGF-β2 signaling pathway and many more. Although many different mechanisms have been proposed for lactate-dependent migration of cancer cells, the whole and complex molecular impact of lactates on tumor metastasis is not entirely understood and needs further studies [51].

In lung cancerogenesis the most important risk factor is cigarette smoking which greatly influences lung inflammation and the redox state [52]. In this study we demonstrated that lung cancer patients had significantly lower total antioxidant capacity compared to control subjects, regardless of circulating glucose concentrations, while parameters related directly to oxidative stress were increased only in patients with elevated glucose levels. However, in our previous study [5], similarly to this one (S2 Table), we found that depletion of the antioxidant defense system in lung cancer was not related to smoking status but rather disease progression. Therefore, we concluded that there are possible factors, other than smoking, which affect redox state in lung cancer. The impact of alteration in glucose metabolism on systemic oxidative stress has been of great interest in many studies [53–55], but such research has not been conducted in populations with lung cancer before. The results obtained in this study confirmed that a high glucose concentration may have a critically negative impact on redox state. Elevated circulating glucose was associated with significantly increased oxidative stress, expressed by TOS and OSI, and this association was additionally intensified by metastasis and former smoking, in which case antioxidant capacity might be weakened. Interestingly, in the subgroup of currently smoking lung cancer patients no correlations between parameters of glucose metabolism and redox status were observed. It might suggest that smoking is a factor in general strongly perturbing redox homeostasis [56], interfering with the relationship between oxidative stress and high glucose concentration. Our speculations concerning interference by smoking of observed associations between redox state and glucose metabolism in lung cancer were further supported by relationships, demonstrated in non-smoking lung cancer patients, between total antioxidant capacity and a parameter suspected of having antioxidant activity–β-hydroxybutyrates [57].

The mechanism of the impact of elevated glucose on redox status has been explained mainly based on diabetes mellitus. As proven in many studies [58–60], reactive oxygen species (ROS) are increased in hyperglycemia. Several mechanisms have been proposed to explain this phenomenon, e.g. higher circulating glucose predisposes towards non-enzymatic glycation of proteins, which show oxidant activity. Additionally, free glucose activates aldose reductase activity and the polyol pathway which decreases the NADPH/NADP+ ratios, as well as protein kinase C, thus in turn increasing oxidative stress by activating mitochondrial NADPH [61].

Interestingly, in the tumor cell milieu, glucose metabolism is altered and promotes the conversion of glucose into substrates needed for biosynthesis and proliferation and moreover is switched towards the metabolic pathway, increasing the synthesis of lactates. Changes in glucose metabolism in tumor cells is uncoupled from oxidative metabolism [62]. As it was experimentally proven, cancer cells inhibit oxidative metabolism of glucose, switching molecular pathways into the Warburg effect which protects against excessive ROS generation and therefore supports metastasis [63]. The positive correlations observed in this study between glucose concentrations and systemic oxidative stress, including in the metastatic sub-group of lung cancer patients, were not in agreement with metabolic associations between high glucose uptake and controlled ROS generation taking place in tumor cells, described by other authors [63,64]. These differences clearly demonstrated between intra- and extracellular compartments in lung cancer, need to be clarified in future research but we speculate that the relationship observed in this study between circulating glucose and systemic oxidative stress resulted from non-enzymatic glycation rather than altered intracellular metabolism in tumor cells. In other studies, observing the subcellular level, associations were related mainly to mitochondria activity, which is predominantly impaired in tumor cells, also in lung cancer ones [65,66]. We envision that identifying these relations at a systemic level may give clinicians a chance to incorporate this knowledge into the routine care of lung cancer patients, for whom systemic disorders in redox status play a significant role in the course of oncological therapy.

The positive correlations found also between fructosamine and parameters of oxidative stress, appear to support the hypothesis that serum protein glycation may predispose to oxidative stress in lung cancer. Fructosamine, as a product of Amadori reaction, is counted among early protein glycation products and after further modifications it formed advanced glycation end products (AGEs). [67]. An increased concentration of these molecules tends to potentiate oxidative stress, and moreover, tobacco smoke is suspected to generate AGEs, regardless of other factors [68]. AGEs were previously shown to be important in the course of lung carcinomas as simple predictors of the outcome of NSCLC patients after curative surgery, and in the case of the impact of a tissue matrix modified by AGE, on the invasive migration of cancer cells [68,69]. Recent evidence suggests that AGEs may act as substrates increasing the expression of receptors for advanced glycation end products (RAGEs) through the activity of NF-kB. Once NF-kB is activated, RAGE expression is continuously increased by a positive feedback loop. Then the nuclear factor erythroid-2-related factor (Nrf2) becomes favourised, activating via RAGE-mediated oxidative stress, which in turn leads to the expression of a disintegrin and metalloproteinase 10 (ADAM10) [70]. An experimental study performed by Guo et al. [71] on NSCLC specimens demonstrated that increasing expression of ADAM10 promoted lung cancer cell migration and invasion via the activation of the transmembrane receptor Notch 1 signaling pathway.

A negative relationship between insulin and antioxidant capacity measured with the ABTS method was also found in other studies, but mostly related to T2DM, where oxidative stress, resulting from a high circulating glucose concentration, induces insulin resistance, which subsequently leads to compensatory higher insulin production and secretion [72,73]. In this study that relationship was observed in non-metastatic lung cancer patients, which suggests that metastasis may disturb this well-known relationship by tumor progression-related mechanisms.

The results obtained in this study demonstrate that systemic parameters related to glucose metabolism (NEFAs, β-hydroxybutyrate) showed antioxidant activity in contrast to parameters directly related to circulating glucose. It remains elusive why in lung cancer patients NEFAs positively correlated with antioxidant capacity and only in metastatic ones was there a switch in metabolic pathways indicating the well-known oxidative properties of NEFAs [74]. In fact, it was proven before that metabolism of NEFAs increases oxidative stress by protein kinase C-dependent activation of NAD(P)H oxidase [75]. Interestingly, to provide the mechanism linking oxidative stress, metastasis in lung cancer and the activation of NAD(P)H oxidase, Liu et al. [76] demonstrated in a recent study that the increased expression of NADPH oxidase-1 (NOX-1) stimulated ROS production and metastasis in non-small cell lung cancer (NSCLC) via the induction of toll-like receptor 4 (TLR-4) signaling. The same mechanism of stimulation of TLR-4 signaling via NEFAs was observed in insulin resistance [77]. Moreover, one of many other possible mechanisms linking the positive correlation between NEFAs and oxidative stress observed in this study in metastatic lung cancer patients could also result from the down-regulated expression of caveolin-1 (Cav-1) [78,79], which in turn independently leads to oxidative stress and defects in the utilization of NEFAs due to the inhibited beta-oxidation of fatty acids [80].

On the other hand, the positive relationship observed in this study between NEFAs and TAS, where the antioxidant pool is determined using ABTS radical scavenging, might result from the specificity of the method and does not exactly reflect, what is going on *in vivo*.

Although we did not observe differences in β-hydroxybutyrate concentrations between metastatic and non-metastatic lung cancer patients or between patients with elevated and normal glucose concentrations, we found that in non-metastatic ones as well as in patients with normal circulating glucose concentrations this ketone body positively correlated with systemic antioxidant capacity. In some studies, it was proven that mild ketosis or supplementation with β-hydroxybutyrate may prevent oxidation by ROS due to an increase in NADH oxidation [81,82]. However, such observations have been made mainly in studies which were not cancer-related, e.g. in brain damage or kidney injuries [82,83]. Interestingly, in metastatic patients we observed a switch in the relationship between β-hydroxybutyrate and redox state parameters. In this sub-group, the ketone body was positively related to OSI. The detailed mechanism of the relationship between ketone bodies and oxidative stress during tumor growth was presented by Pavlides et al. [80], whose study concerned the autophagic tumor stroma model of cancer. In this study, a high level of β-hydroxybutyrate was related to oxidative stress due to its overproduction during mitochondrial dysfunctions [80], which were also observed in lung cancer [65,66]. Moreover, high β-hydroxybutyrate concentration stimulated tumor progression and metastasis as a stromal metabolite for tumor cell growth [80]. In a study by Pavlides et al. [80], a higher concentration of β-hydroxybutyrate was the result of the overexpression of enzymes related to ketone production following the loss of caveolin-1 expression in Cav-1 (-/-) mouse lung tissue, as well as in human tumor stroma. Elevated β-hydroxybutyrate was in association with oxidative stress as a paracrine energy source transported by monocarboxylate transporters (MCT) from tumor stromal cells to epithelial cells, where it could directly enter the tricarboxylic acid cycle (TCA) as mitochondrial fuel to produce ATP [80]. Loss of caveolin-1 expression was observed in several studies concerning lung cancer [78,79], thus supporting our insight into the mechanisms of the relationships observed in our study.

### Limitations of the study

Limitation of the study should be considered when interpreting the data. Since the presented associations may be different in the tumor milieu, similar studies should be performed additionally in tissues of lung tumors. Moreover, lung cancer is partially determined by genetic polymorphism, so it might be interesting to evaluate the possible impact of variations in antioxidant genes and genes related to glucose metabolism on redox status changes to find a genetic basis linking these two phenomena. Further investigations are warranted to confirm these findings and to explore the underlying mechanisms in detail, especially genetic ones, and to identify new pharmacological perspectives of lung cancer treatment.

## Conclusions

**Fig 1:**
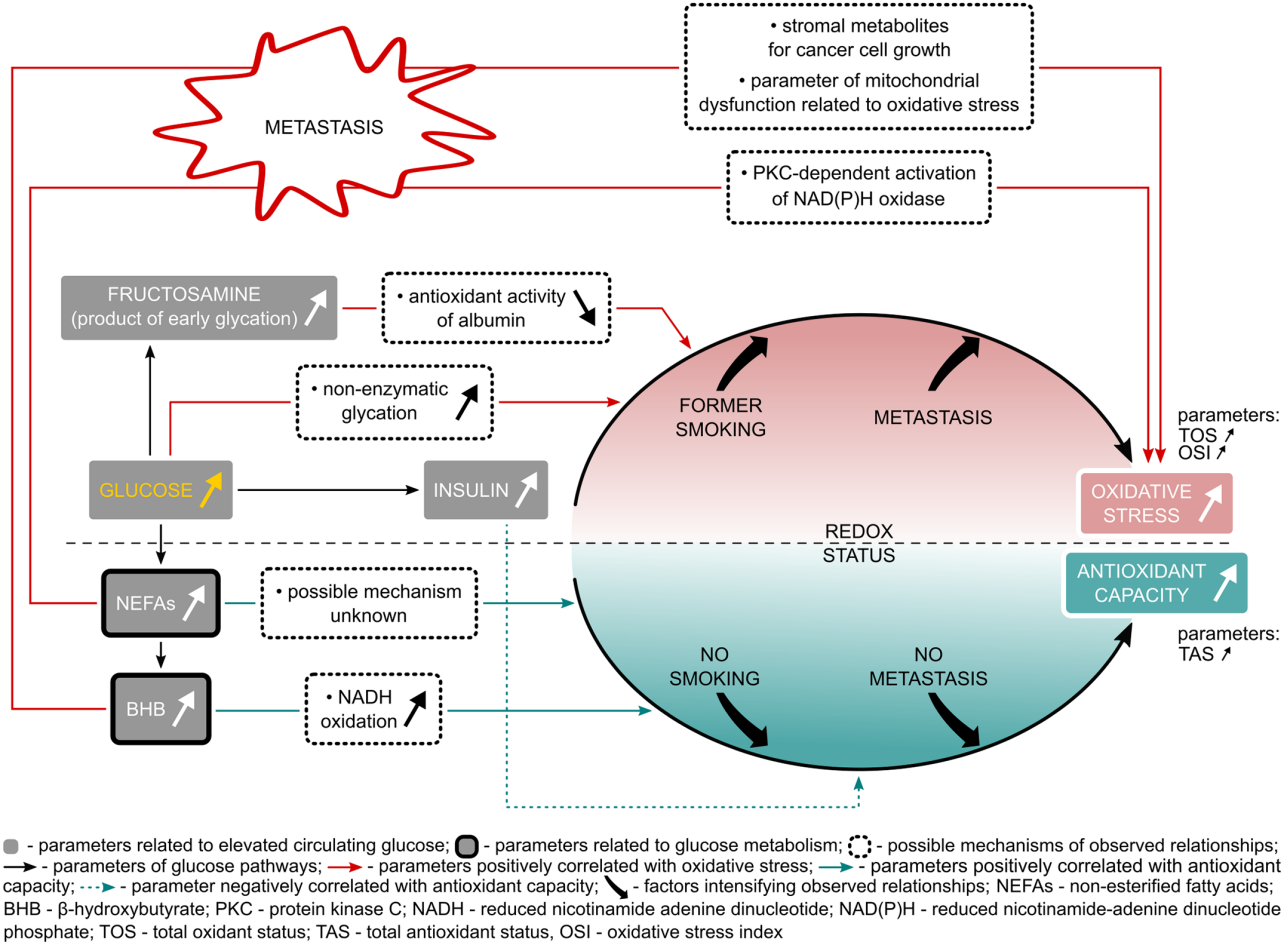
Relationships with possible mechanisms between glucose metabolism and redox status in lung cancer patients.

Based on the presented results, we demonstrated that parameters related to circulating glucose or non-enzymatic glycation are correlated with oxidative stress (TOS and OSI), while metabolites such as β-hydroxybutyrate and NEFAs are linked with TAS in lung cancer patients. Metastasis prevalence and smoking seem to influence these correlations. However, detailed mechanisms of these relationships require further research, particularly considering the surprising positive correlation between NEFAs and TAS (Fig 1). The correlations and proposed mechanisms presented in this study may serve as a basis for future studies which could include lowering glucose levels or improving redox activity in lung cancer patients to support the positive outcome of them.

Parameters related to circulating glucose were positively correlated with oxidative stress and observed relationships were stronger in former smokers and metastatic lung cancer patients. Insulin was negatively correlated with TAS. Parameters related to glucose metabolism positively correlated with TAS and these correlations were stronger in non-smokers and non-metastatic patients. In metastatic ones, these parameters were positively correlated with parameters of oxidative stress (TOS, OSI).

## Supporting information

S1 Table Biochemical variables related to glucose metabolism and redox status parameters in lung cancer patients with different clinical stages of disease [median (min − max)]. (DOCX)

S2 Table Biochemical variables related to glucose metabolism and redox status parameters in lung cancer patients with different smoking status [median (min − max)]. (DOCX)

S1 File Original file of data used in analyzes. (XLSX)
