# Supplementary material for: Systemic redox status in lung cancer patients is related to altered glucose metabolism
Source: PLoS One. 2018 Sep 20;13(9):e0204173. doi: 10.1371/journal.pone.0204173 (PMC6147499; doi:10.1371/journal.pone.0204173)
Supplement: S1 Table — (DOCX) [file pone.0204173.s001.docx]

**Supplementary table 1. Systemic parameters related to glucose metabolism and redox status in lung cancer patients with different clinical stage of disease [median (min – max)]**

| **Parameters** | **LC**  **Stage I** | **LC**  **Stage II** | **LC**  **Stage III** | **LC**  **Stage IV** |
| --- | --- | --- | --- | --- |
|  | **parameters related to glucose metabolism** | | | |
| **GLC [mg/dL]** | 126.0 (57.0 – 296.0) | 94.0 (84.0 – 126.0) | 111.0 (71.0 – 201.0) | 91.0 (64.0-230.0) |
| **INS [µIU/mL]** | 19.9 (1.16 – 112.7) | 21.4 (7.59 – 82.5) | 15.2 (6.41 – 30.7) | 9.29 (3.13 – 89.5) |
| **HOMA-IR [arbitrary unit]** | 5.79 (0.24 – 68.3) | 5.25 (1.76 – 24.0) | 4.44 (1.31 – 10.5) | 2.58 (0.49 – 41.3) |
| **C-PEP [ng/mL]** | 4.18 (0.13 – 12.2) | 2.73 (0.84 – 11.73) | 4.14 (1.00 – 11.7) | 2.91 (0.90 – 9.36) |
| **ΒHB [mmol/L]** | 0.10 (0.02 – 1.19) | 0.22 (0.04 – 0.77) | 0.08 (0.03 – 0.53) | 0.10 (0.03 – 1.05) |
| **FA [µmol/1g ALB]** | 8.62 (6.38 – 14.5) | 7.64 (6.37 – 9.68) | 8.96 (6.15 – 11.3) | 8.54 (6.23 – 17.1) |
| **LACT [mmol/L]** | 2.39 (0.95 – 4.67) | 2.42 (1.95 – 3.33) | 2.4 (1.18 – 3.67) | 3.13 (1.50 – 4.48) |
| **NEFAs [mmol/L]** | 0.47 (0.27 – 1.46) | 0.48 (0.45 – 0.51) | 0.55 (0.25 – 1.04) | 0.56 (0.28 – 0.98) |
|  | **parameters related to redox status** | | | |
| **TAS [mmol Trolox equiv./L]** | 1.66 (1.18 – 2.55) | 1.57 (1.35 – 1.74) | 1.75 (0.96 – 2.01) | 1.57 (1.12 – 2.01) |
| **TOS [µmol H_2_O_2_ equiv./L]** | 3.71 (0.89 – 74.8) | 3.11 (1.81 – 47.6) | 4.56 (1.05 – 45.01) | 3.20 (1.00 – 25.5) |
| **OSI [arbitrary unit]** | 2.57 (0.66 – 51.9) | 1.83 (1.24 – 28.0) | 2.35 (0.78 – 25.3) | 1.89 (0.74 – 17.6) |

LC – lung cancer patients; CS – control subjects; GLC – glucose concentration; INS – insulin concentration; HOMA-IR – homeostasis model assessment – insulin resistance; C-PEP – C-peptide concentration, BHB – β-hydroxybutyrate concentration; FA – fructosamine concentration; LACT - lactate concentration; NEFAs – non-esterified fatty acids concentration; TAS – total antioxidant status; TOS – total oxidant status; OSI –oxidant status index; no differences were observed using post-hoc tests
