# Supplementary material for: Systemic redox status in lung cancer patients is related to altered glucose metabolism
Source: PLoS One. 2018 Sep 20;13(9):e0204173. doi: 10.1371/journal.pone.0204173 (PMC6147499; doi:10.1371/journal.pone.0204173)
Supplement: S2 Table — (DOCX) [file pone.0204173.s002.docx]

**Supplementary table 2. Systemic parameters related to glucose metabolism and redox status in lung cancer patients with different smoking status [median (min – max)]**

| **Parameters** | **Non-smoking LC**  **(n=49)** | **Former smoking LC**  **(n=40)** | **Smoking LC**  **(n=28)** |
| --- | --- | --- | --- |
| **parameters related to glucose metabolism** | | | |
| **GLC [mg/dL]** | 101.0 (57.0 – 286.0) | 112.0 (73.0 – 296.0) | 91.5 (64.0 – 145.0) |
| **INS [µIU/mL]** | 21.4 (4.11 – 115.5) | 15.3 (3.35 – 110.3) | 9.46 (3.13 – 50.0) |
| **HOMA-IR [arbitrary unit]** | 6.13 – 0.79 – 81.6) | 5.04 (0.64 – 45.7) | 2.06 (0.49 – 14.1) |
| **C-PEP [ng/mL]** | 4.77 (0.51 – 12.3) | 3.34 (0.25 – 12.2) | 2.88 (0.79 – 9.49) |
| **ΒHB [mmol/L]** | 0.1 (0.03 – 1.19) | 0.11 (0.02 – 2.57) | 0.07 (0.03 – 1.11) |
| **FA [µmol/1g ALB]** | 8.50 (6.15 – 11.17) | 8.38 (6.37 – 17.1) | 8.73 (6.23 – 14.0) |
| **LACT [mmol/L]** | 2.84 (0.74 – 32.2) | 2.55 (1.2 – 4.35) | 2.87 (1.18 – 4.48) |
| **NEFAs [mmol/L]** | 0.60 (0.29 – 1.03) | 0.60 (0.29 – 1.46) | 0.60 (0.25 – 3.30) |
| **parameters related to redox status** | | | |
| **TAS [mmol Trolox equiv./L]** | 1.64 (0.96 – 2.55) | 1.45 (1.12 – 2.34) | 1.57 (1.18 – 2.01) |
| **TOS [µmol H_2_O_2_ equiv./L]** | 3.67 (1.00 – 47.6) | 5.40 (0.89 – 74.8) | 4.60 (1.05 – 41.7) |
| **OSI [arbitrary unit]** | 2.40 (0.74 – 28.0) | 3.98 (0.67 – 51.9) | 2.89 (0.78 – 31.1) |

LC – lung cancer patients; CS – control subjects; GLC – glucose concentration; INS – insulin concentration; HOMA-IR – homeostasis model assessment – insulin resistance; C-PEP – C-peptide concentration, BHB – β-hydroxybutyrate concentration; FA – fructosamine concentration; LACT - lactate concentration; NEFAs – non-esterified fatty acids concentration; TAS – total antioxidant status; TOS – total oxidant status; OSI –oxidant status index; no differences were observed using post-hoc tests
